# Enterococcal Infections the First Year after Liver Transplantation—A Prospective Cohort Study

**DOI:** 10.3390/microorganisms9081740

**Published:** 2021-08-15

**Authors:** Daniel B. Rasmussen, Dina L. Møller, Andreas D. Knudsen, Andreas A. Rostved, Jenny D. Knudsen, Allan Rasmussen, Susanne D. Nielsen

**Affiliations:** 1 Department of Infectious Diseases, Rigshospitalet, University of Copenhagen, 2100 Copenhagen, Denmark; Daniel.braeuner.rasmussen@regionh.dk (D.B.R.); Dina.leth.moeller@regionh.dk (D.L.M.); Andreas.dehlbaek.knudsen@regionh.dk (A.D.K.); 2 Department of Cardiology, Rigshospitalet, University of Copenhagen, 2100 Copenhagen, Denmark; 3 Department of Surgical Gastroenterology and Transplantation, Rigshospitalet, University of Copenhagen, 2100 Copenhagen, Denmark; andreas.arendtsen.rostved@regionh.dk (A.A.R.); Allan.Rasmussen@dadlnet.dk (A.R.); 4 Department of Clinical Microbiology, Rigshospitalet, University of Copenhagen, 2100 Copenhagen, Denmark; Inge.Jenny.Dahl.Knudsen@regionh.dk; 5 Department of Clinical Medicine, University of Copenhagen, 2100 Copenhagen, Denmark

**Keywords:** liver transplantation, enterococcal infections, bloodstream infections, biliary tract infection, bacteremia, cholangitis, antibiotic resistance

## Abstract

This study aimed to investigate the incidence of enterococcal infections and determine risk factors associated with enterococcal bloodstream infection (BSI) within the first year post-liver transplantation (LTx). We included 321 adult liver transplant recipients transplanted from 2011 to 2019 in a prospective cohort study. Cumulative incidence of enterococcal infections and risk factors associated with BSI were investigated in a competing risk model and time-updated Cox models, respectively. A total of 223 enterococcal infections were identified in 89 recipients. The cumulative incidences of first enterococcal infection and first enterococcal BSI were 28% (95% CI (23–33)) and 11% (CI (7–14)), respectively. Risk factors associated with enterococcal BSI were previous infections in the biliary tract (HR, 33; CI (15–74); *p* < 0.001), peritoneum (HR, 8.1; CI (3–23); *p* < 0.001) or surgical site (HR, 5.5; CI (1.4–22); *p* = 0.02), recipient age (HR per 10 years increase, 1.2; CI (1.03–1.6); *p* = 0.03), and cold ischemia time (HR per one hour increase, 1.2; CI (1.1–1.3); *p* < 0.01). Enterococcal infections are highly prevalent the first year post-LTx, and recipients with enterococcal infections in the biliary tract, peritoneum, or surgical site are at increased risk of BSI. These findings may have implications for the choice of empiric antibiotics early post-LTx.

## 1. Introduction

Bacterial infections post-LTx are common, with incidence rates as high as 30% in the first month [3]. Among bacterial infections in liver transplant recipients, *Enterococcus* spp. are particularly common [4,5,6]. Enterococci are intrinsically resistant to some of the commonly used antibiotics, and with the rapid spread of extrinsic resistance to glycopeptide and oxazolidinone antibiotics, the emergence of vancomycin-resistant (VRE) and linezolid-resistant (LRE) enterococci has become a concern [7]. The epidemiology of enterococcal infections differs between different transplant units, with the proportion of VRE ranging from 4% to 11% of enterococcal infections [8,9,10].

The short-term prognosis after liver transplantation (LTx) has improved dramatically within the last five decades. This is primarily explained by improvements in surgical techniques and immunosuppressive treatment [1]. However, immunosuppressive treatment is associated with an increased risk of infections that contributes to morbidity and mortality in liver transplant recipients [1,2].

Importantly, focal enterococcal infections may disseminate and give rise to enterococcal bloodstream infection (BSI) that are associated with increased morbidity and mortality, and the site of focal infections may be a specific risk factor [10,11,12,13]. However, risk factors for the progression to enterococcal BSI remain uncertain.

In a large nationwide prospective study of all adult liver transplant recipients in Denmark during the period 2011–2019, we aimed to investigate the incidence and characteristics of enterococcal infections and to determine risk factors for progression to enterococcal BSI in adult liver transplant recipients.

## 2. Materials and Methods

### 2.1. Study Design and Participants

We prospectively included all adult mono-organ first-time LTx recipients transplanted from 1 January 2011 to 1 April 2019 at Rigshospitalet, Copenhagen University Hospital, which is the only center for liver transplantation in Denmark.

Recipients were identified by a 10-digit Civil Personal Registration (CPR) number that is provided to all persons who live in Denmark. Recipient-related variables were collected from patient records, and graft-related variables were retrieved from ScandiaTransplant, the organ exchange organization in Scandinavia [14].

For calculation of the MELD score, we used serum bilirubin, serum creatinine, and international normalized ratio (INR) collected immediately prior to transplantation [15] and the formula: 3.78 × ln(serum bilirubin mg/dl)  +  11.2 × ln(INR)  +  9.57 × ln(creatinine mg/dl)  +  6.43. If treated with renal or liver replacement therapy twice within the last week pre-LTx, creatinine levels were fixed at 4 mg/dl [15].

### 2.2. Immunosuppressive Regimes and Antibiotic Prophylaxis

Standard immunosuppression included mycophenolate mofetil, prednisolone, and tacrolimus guided by trough levels. Furthermore, in accordance with local guidelines, basiliximab and/or cyclosporin were used in recipients with type 1 diabetes mellitus, acute transplantation, or renal function with glomerular filtration rate < 70 mL/min.

Standard antibiotic prophylaxis post-LTx was 2 g meropenem every 8 h for the first 5 days post-LTx. Guidelines for *cytomegalovirus* (CMV), anti-fungal, and *Pneumocystis jirovecii* (PCP) prophylaxis in our center are shown in Supplementary Materials.

### 2.3. Microbiology

Microbiology data were retrieved from the Danish Microbiology Database (MiBa), which has complete coverage of all samples both from general practice and hospitals processed in Departments of Clinical Microbiology in Denmark since 2010 [16]. We retrieved all isolates of enterococci from 1 January 2011 to 1 April 2020.

Enterococcal samples were considered as an infection if meeting the CDC/NHSN surveillance definitions and criteria [17]. We defined the following focal sites of infection: biliary tract, urinary tract, peritoneum, abscess, surgical sites, and others. Multiple enterococcal samples from the same site and with the same species were considered part of the same infection if isolated within 14 days [17].

Enterococcal bloodstream infection was defined as any finding of enterococci in blood. When the species of a BSI matched that of an infection in one or more focal sites, the BSI was interpreted as secondary if occurring within 3 days before to 14 days after the focal infection [17].

### 2.4. Follow-Up

We followed liver transplant recipients from date of transplantation until one-year post-LTx, retransplantation, or death, whichever came first. Enterococcal infections were divided into three time periods: first month, 2–6 months, and 7–12 months post-LTx [18]. Infections occurring after the first year post-LTx were not reported.

### 2.5. Statistical Analysis

Categorical data are presented as percentages, and continuous data are presented as medians with interquartile ranges (IQR). The cumulative incidence of first infection was calculated using the Aalen–Johansen estimator, with death and retransplantation as competing risks. Pre-transplant risk factors for enterococcal BSI within the first year post-LTx were investigated in Cox proportional hazard models, adjusted for age, sex, and diabetes. To investigate, if post-transplant enterococcal infections stratified by the focal site of infection predicted enterococcal BSI, we performed a Cox proportional hazard model with the site of infection as time-dependent covariates, adjusted for age, sex, and diabetes. For this analysis, recipients changed status with each enterococcal infection, apart from BSI, from “no enterococcal infection” to “enterococcal infection”. The status remained for 14 days, at which time it changed back to “no enterococcal infection”. This allowed us to account for the temporal relationship between enterococcal infections and BSI. No infections at the category other sites progressed to bloodstream infection; thus, the model was not able to converge, and other sites were not included in the model. All analyses were conducted in the statistical software R version 3.6.1 (R Foundation for Statistical Computing, Vienna, Austria) [19].

## 3. Results

### 3.1. Characteristics of the Liver Transplant Recipients

We included all 321 adult, mono-organ, first-time liver transplant recipients transplanted in Denmark from 1 January 2011 to 1 April 2019 (Table 1). The average follow-up was 338 days (range 3–365). Of the 321 included recipients, 23 (7%) recipients died and 14 (4%) were re-transplanted within the first year post-LTx.

### 3.2. Characteristics of the Enterococcal Infections

In 89 of the 321 recipients (28%), a total of 293 enterococcal samples were identified during the study period. Of these, 70 samples were collected within the 14-day repeated time frame, leaving 223 unique infections in 89 recipients. In total, 43 (48%) recipients had one infection, whereas 46 (52%) had multiple.

**Figure 1:**
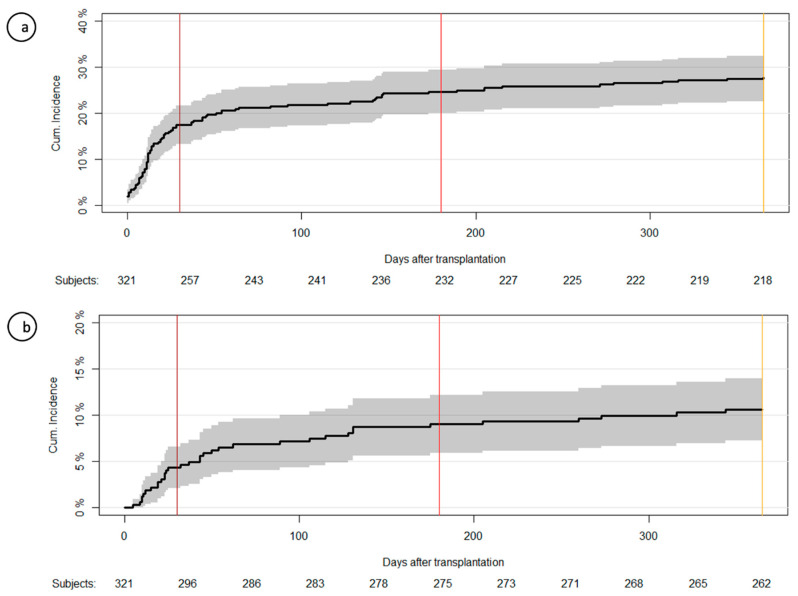
Cumulative incidence of first enterococcal infection and first bloodstream infections. (**a**) Cumulative incidence curve of first enterococcal infection post-LTx as a function of days post-LTx. (**b**) Cumulative incidence curve of first enterococcal bloodstream infections post-LTx as a function of days post-LTx. Vertical lines: At day 30 (brown), 180 (red), and 365 (yellow).

The cumulative incidence of at least one infection was 28% in the first year post-LTx (95% confidence interval (CI), 23–33) (Figure 1a). The most common enterococcal species were *E. faecium* and *E. faecalis*, found in 172 (77%) and 40 (18%) of the infections, respectively. The cumulative incidence of enterococcal infections stratified by time periods post-LTx and site of infection is shown in Table 2.

### 3.3. Antibiotic Resistance Pattern

Antibiotic susceptibility profiles were available for 220 of the 223 infections (99%), with 24 (12%) and 11 (6%) infections lacking tests for linezolid and vancomycin resistance, respectively (Table 2). Of the 185 infections with data on vancomycin susceptibility, 32 enterococcal infections (16%) in 18 recipients (6%) were caused by vancomycin-resistant enterococci (VRE). Furthermore, one enterococcal infection (0.5%) was caused by linezolid-resistant enterococci (LRE), and four (2%) infections in four recipients (1%) were caused by combined linezolid- and vancomycin-resistant enterococci (LVRE).

### 3.4. Enterococcal Bloodstream Infections

The cumulative incidence of at least one enterococcal BSI in the first year post-LTx was 11% (95% CI, 7–14) (Figure 1b). The cumulative incidence of first BSI was 4% within the first month (95% CI, 2–7) and 9% within the first 6 months post-LTx (95% CI, 6–12). The 49 enterococcal BSIs consisted of 38 *E. faecium* (78%), 10 *E. faecalis* (20%), and 1 *E. gallinarum* (2%).

In total, 21 (43%) bloodstream infections could be considered secondary to focal enterococcal infections, most frequently after biliary tract infections (47%) (Table 3). Of the 32 recipients who had biliary tract infection, 10 recipients (31%) developed a secondary BSI.

### 3.5. Risk Factors

**Figure 2:**
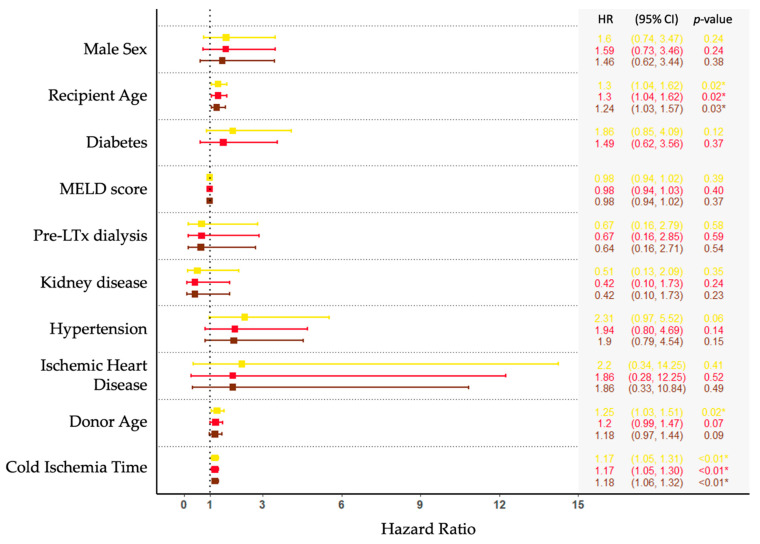
Cox hazard ratios of recipient- and graft-characteristics. Forest plots represent the unadjusted (yellow), adjusted for age and sex (red), and fully adjusted (brown) models. Note: One person may experience several bloodstream infections. Recipient Age, age at the time of transplantation, continuous variable measured in per 10 years older; MELD score, continuous variable measured in the value of Model for End-Stage Liver Disease; Donor Age, age at the time of graft removal, continuous variable measured in per 10 years older; Cold Ischemia Time, time between the chilling and removal of donor liver and the time of restored blood supply in recipient, continuous variable measured in hours. The asterisk (*) represents *p* < 0.05.

Recipient- and graft-related characteristics associated with enterococcal BSI are shown in Figure 2. When adjusting for age, sex, and diabetes, older recipient age (HR per 10 years older, 1.2; CI (1.03–1.6); *p* = 0.03) and longer cold ischemia time (HR per one hour longer, 1.2; CI (1.1–1.3); *p* < 0.01) were associated with increased risk of enterococcal BSI.

**Figure 3:**
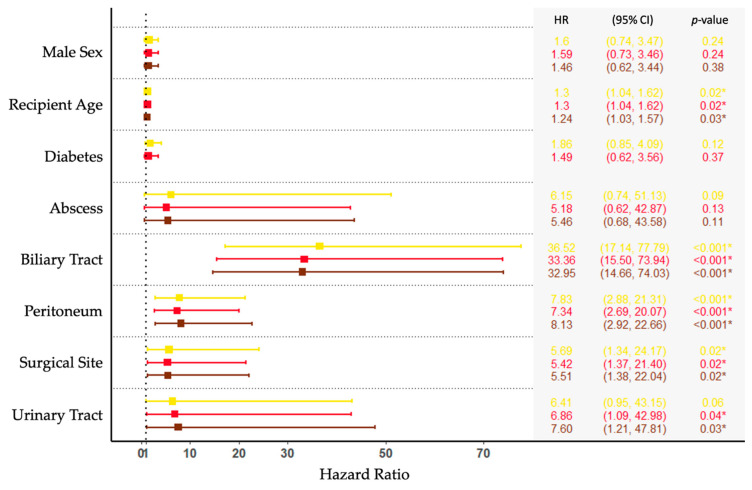
Time-updated Cox hazard functions of site of focal enterococcal infection. Forest plots represent the unadjusted (yellow), adjusted for age and sex (red), and adjusted for age, sex, and diabetes (brown) models. Note: One person may experience several bloodstream infections. All sites were time-updated variables contributing to the risk of enterococcal bloodstream infection 3 days before to 14 days after the focal enterococcal infection. The asterisk (*) represents *p* < 0.05.

The hazard ratios for the development of a BSI depending on the sites of the previous focal infection, adjusted for recipient age, sex, and diabetes, are shown in Figure 3*.* When adjusting for age, sex, and diabetes, sites of focal infections associated with an increased risk of enterococcal secondary BSI were biliary tract (HR, 33; CI (15–74); *p* < 0.001), peritoneum (HR, 8.1; CI (3–23); *p* < 0.001), surgical site (HR, 5.5; CI (1.4–22); *p* = 0.02), and urinary tract (HR, 7.6; CI (1.2–48); *p* = 0.03). Of note, urinary tract infection was not associated with an increased risk of secondary BSI in the unadjusted model.

## 4. Discussion

In this prospective nationwide study of all liver transplant recipients in Denmark during the period 2011–2019, we determined the incidence of enterococcal infections and risk factors for enterococcal BSI. In total, over a quarter of the recipients developed at least one enterococcal infection in the first year post-LTx, and enterococcal BSIs occurred in over one in ten of the recipients. Vancomycin-resistant enterococci were frequent, while linezolid-resistant enterococci were rare. The risk factors associated with the development of enterococcal BSI were older recipient age, longer cold ischemia time, and previous infections in the biliary tract, peritoneum, and surgical site.

Our estimate of the incidence of enterococcal infections in the first year after transplantation is similar to reports from The Swiss Transplant Cohort Study, in which 20% of the liver transplant recipients experienced an enterococcal infection within the first 6 months post-LTx [6]. We found that enterococcal infections were most frequent in the first and second–sixth months post-LTx. This is in line with other studies that have reported enterococcal infections to be frequent within the first month post-LTx [6,13]. Likewise, enterococcal BSIs were most frequent within the first 6 months post-LTx. The same tendency has been reported before [4], including another study at our center investigating BSI in pediatric solid organ transplant recipients, including LTx, where the incidence rate of BSI appeared highest in the early post-transplantation phase [20], possibly reflecting a higher risk due to the intensified immunosuppression early post-transplant and surgical complications.

We investigated risk factors for the development of enterococcal BSI. Of the investigated graft- and recipient-related variables, older recipient age and longer cold ischemia time predicted BSI. Likewise, The Swiss Transplant Cohort Study found older recipient age to be significantly associated with the risk of enterococcal infections [6], whereas others have found no significant association with enterococcal bacteremia [13]. Prolonged cold ischemia time is usually not regarded as a risk factor for BSIs. However, cold ischemia time has recently been associated with early recurrent BSI post-LTx [21]. A possible mechanism may be impaired regenerative ability, leading to longer ICU stays and increased use of invasive devices, thus, indirectly increasing the risk of BSI [22,23].

We found previous infections in the biliary tract and peritoneum to be common primary sites of secondary enterococcal BSIs, whereas urinary tract and surgical site were less frequent and were associated with lower hazard ratios. Unlike our results, a recent study found urinary tract and surgical site to be the most common sites of secondary bacteremia infections, whereas, intra-abdominal infections causing secondary bacteremia were less frequent [12]. However, the study included all BSI—Gram-positive and Gram-negative—in a tertiary care hospital, making direct comparison difficult due to the differences in the patient composition in the investigated cohorts and the causative pathogens.

Importantly, we found biliary tract infections to be associated with a high risk of secondary enterococcal BSI post-LTx, and 31% of recipients with enterococcal biliary tract infections developed secondary BSIs. Biliary tract infections have previously been associated with bacteremia [24] and were found to be associated with bile leak after pancreaticoduodenectomy [25]. A possible mechanism may be gelatinase activity of *E. faecalis* degrading collagen, resulting in a prolonged healing process in such infections [25,26]. Thus, enterococcal biliary tract infection could be a marker of poor healing of the biliary anastomosis post-LTx, creating a possible pathway to the bloodstream. However, only 20% of biliary tract infections progressing to enterococcal BSIs were caused by *E. faecalis*, whereas *E. faecium* caused the remaining secondary BSIs in our cohort. Furthermore, this association might reflect that biliary tract infections are treated less vigilantly or less effectively compared with other sites of enterococcal infection.

Previous studies have found VRE infection rates of 4%–11% post-transplantation [8,9]; similarly, we found that 6% of recipients had VRE infections, with the highest frequency of VRE in other enterococcal species and no VRE in *E. faecalis* infections. Likewise, a study on enterococcal BSIs post-LTx found that VRE was frequently *E. faecium*, whereas *E. faecalis* infections were all susceptible to vancomycin [13]. This highlights the importance of a differentiated approach to the antibiotic treatment of enterococcal infections, with a lower threshold for additional treatment in other enterococcal species and *E. faecium*. Based on our findings, one might think piperacillin/tazobactam would be a reasonable choice for prophylactic regimen post-transplantation. However, our results should be interpreted with caution since we only investigated enterococcal infections, and a previous study from our center found that liver transplant recipients have diverse bacterial infections post-LTx [20]. Furthermore, prophylaxis treatment should always be based on local resistance patterns.

The major strengths of our study are the prospective design and large study population. Furthermore, all liver transplantations in Denmark are performed at Rigshospitalet. Lastly, we had a complete nationwide follow-up of the recipients, ensured by the CPR registry, and the microbiological data were ensured by the MiBa database, which covers all microbiological samples performed in Denmark. Thus, recipients are only lost to follow-up if they migrate out of the country.

A limitation of this study is that we did not assess antibiotic treatments or neutropenia post-transplantation. Furthermore, poly-microbial infections were not addressed.

## 5. Conclusions

In conclusion, enterococcal infections are frequent in the first year post-LTx, occurring in one out of four liver transplant recipients, and enterococcal bloodstream infection occurred in 11% of recipients. Important risk factors for the development of enterococcal bloodstream infection were older recipient age, longer cold ischemia time, and previous infections in the biliary tract, peritoneum, and surgical site. VRE infections were seen in 6% of the recipients, whereas LRE infections were rare. Vancomycin resistance was primarily found in *E. faecium* infections, whereas no vancomycin resistance was detected in *E. faecalis*. These findings may have implications for the choice of empiric antibiotics early post-LTx.

## Figures and Tables

**Table 1:** Cohort demographics.

Total Number of Recipients		321
Age at Transplantation, Median (IQR)		50 (42–57)
Gender, Male (%)		186 (58)
Pre-LTx MELD ^1^ score, median (IQR)		13.8 (9–19)
Underlying liver disease of recipients, n (%)	Primary sclerosing cholangitis	89 (28)
	Alcoholic liver disease	45 (14)
	Primary biliary cholangitis	35 (11)
	Cryptogenic cirrhosis	28 (9)
	Hepatocellular carcinoma	23 (7)
	Hepatitis C	19 (6)
	Fulminant hepatic failure	18 (6)
	Autoimmune hepatitis	12 (4)
	Other ^2^	52 (16)
Comorbid conditions of recipients, n (%)	Diabetes mellitus	64 (20)
	Essential hypertension	63 (20)
	Kidney disease	18 (6)
	Ischemic heart disease	6 (2)
	HIV infection	0 (0)

^1^ MELD, Model for End-Stage Liver Disease; ^2^ Other included polycystic liver disease, hepatitis B, alpha-1 antitrypsin deficiency, amyloidosis, Budd–Chiari Syndrome, progressive familial intrahepatic cholestasis, etc.

**Table 2:** Characteristics of enterococcal infections.

**Enterococcal Infections, n (%)**		***E. faecium***	***E. faecalis***	**Other Spp. ^1^**	**Total**	**Cumulative Incidence, % (95% CI)**
		172 (77)	40 (18)	11 (5)	223	28 (23–33)
Time period of infection, n (%)	1st month	66 (76)	16 (18)	5 (6)	87	15 (11–19)
	2nd–6th month	87 (84)	14 (13)	3 (3)	104	12 (8–15)
	7th–12th month	19 (59)	10 (31)	3 (10)	32	6 (3–9)
Site of infections, n (%)	Biliary Tract	41 (72)	14 (25)	2 (3)	57	8 (5–11)
	Peritoneum	40 (89)	4 (9)	1 (2)	45	11 (7–14)
	Surgical site	19 (79)	2 (8)	3 (13)	24	6 (3–9)
	Urinary tract	9 (60)	5 (33)	1 (7)	15	4 (2–6)
	Abscess	10 (77)	3 (23)	0 (0)	13	4 (2–6)
	Other sites ^2^	15 (75)	2 (10)	3 (15)	20	6 (3–8)
	Bloodstream	38 (78)	10 (20)	1 (2)	49	11 (7–14)
Antibiotic resistance ^3^, n (%)	Ampicillin	166 (98)	0 (0)	3 (2)	169	-
	Vancomycin	28 (88)	0 (0)	4 (12)	32	-
	Linezolid	4 (80)	1 (20)	0 (0)	5	-

^1^ Other spp. included: 2 *E. gallinarum*, 2 *E. classeliflavus*, 1 *E. hirae*, 1 *E. raffinosus*, and 5 findings of unknown species. ^2^ Other sites included: 12 respiratory tract, 6 soft tissue, and 2 endometrial infection. ^3^ Ampicillin, vancomycin and linezolid resistance patterns were available in 99%, 94%, and 88% of the infections, respectively.

**Table 3:** Characteristics of enterococcal bloodstream infections.

		All Enterococcal BSI (n = 49)
Species of BSI	*E. faecium*	38 (78%)
	*E. faecalis*	10 (20%)
	Other spp. ^1^	1 (2%)
Site of infections, n (%)	Biliary tract	10 (47%)
	Peritoneum	5 (24%)
	Surgical site	4 (19%)
	Urinary tract	1 (5%)
	Abscess	1 (5%)
	Other sites	0 (0%)
Secondary BSI (% of all enterococcal BSI)		21 (43%)
Species of secondary BSI	*E. faecium*	19 (90%)
	*E. faecalis*	2 (10%)
	Other spp.	0 (0%)

^1^ Other spp. included one *E. gallinarum*.

## Data Availability

The data presented in this study are available to be seen in person at our institution upon request. The data are not publicly available due to the data protection law in Denmark.

## Supplementary Materials

The following are available online at https://www.mdpi.com/article/10.3390/microorganisms9081740/s1, Text S1: *Cytomegalovirus*, anti-fungal, and *Pneumocystis jirovecii* prophylaxis.
