# Supplementary material for: Enterococcal Infections the First Year after Liver Transplantation—A Prospective Cohort Study
Source: Microorganisms. 2021 Aug 15;9(8):1740. doi: 10.3390/microorganisms9081740 (PMC8400967; doi:10.3390/microorganisms9081740)
Supplement: Supplementary file 1 [file microorganisms-09-01740-s001.zip › microorganisms-1316825-supplementary.pdf]

Enterococcal infections the first year after liver transplantation – a prospective cohort study

Daniel Bräuner Rasmussen, M.B.B.S[a] ; Dina Leth Møller, MD[a] ; Andreas Dehlbæk Knudsen, MD[a,b] ; Andreas Arendtsen Rostved, MD, PhD[c] ; Inge Jenny Dahl Knudsen, MD, DMSc[d] ; Allan Rasmussen, MD[c] ; Susanne Dam Nielsen, Professor, MD, DMSc[a,c,e*.]

[a] Department of Infectious Diseases, Rigshospitalet, University of Copenhagen, Copenhagen, Denmark.

Daniel.braeuner.rasmussen@regionh.dk

Dina.leth.moeller@regionh.dk

aknu0050@regionh.dk

sdn@dadlnet.dk

[b] Department of Cardiology, Rigshospitalet, University of Copenhagen, Copenhagen, Denmark.

[c] Department of Surgical Gastroenterology and Liver Transplantation, Rigshospitalet, University of Copenhagen, Copenhagen, Denmark.

andreas.arendtsen.rostved@regionh.dk

Allan.Rasmussen@dadlnet.dk

[d] Department of Clinical Microbiology, Rigshospitalet, University of Copenhagen, Copenhagen, Denmark.

Inge.Jenny.Dahl.Knudsen@regionh.dk

[e] Department of Clinical Medicine, University of Copenhagen, Copenhagen, Denmark.

* Corresponding author at: Viro-immunology Research Unit, Department of Infectious Diseases 8632, Rigshospitalet, University of Copenhagen, Copenhagen, Denmark.

E-mail address: sdn@dadlnet.dk, Phone: (+45) 35 45 0859, Fax: (+45) 35456648

Postal Address: Viro-immunology Research Unit, Department of Infectious Diseases 8632, Rigshospitalet, University of Copenhagen, Blegdamsvej 9B, DK-2100 Copenhagen Ø, Denmark.

Supplementary material

Supplementary Text S1

Cytomegalovirus, anti-fungal and *Pneumocystis jirovecii* prophylaxis

CMV management in the cohort is explained elsewhere [27]. To summarise, preemptive PCR screening and valganciclovir prophylaxis was used for selected recipients based on CMV IgG serostatus. Furthermore, recipients received 6 months of trimethoprim-sulfamethoxazole prophylaxis for *Pneumocystis jirovecii* infection. Additional antifungal prophylaxis was administered in selected cases, including recipients undergoing prolonged surgery time, retransplantation, additional surgery post-LTx, massive blood loss, continuous renal replacement therapy, fulminant hepatitis, or CMV infection treated with Micafungin or Anidulafungin.
